# The Role and Value of Professional Rapid Testing of Acute Respiratory Infections (ARIs) in Europe: A Special Focus on the Czech Republic, Poland, and Romania

**DOI:** 10.3390/diagnostics14060631

**Published:** 2024-03-16

**Authors:** Pavel Drevinek, Robert Flisiak, Roxana Nemes, Katya A. Nogales Crespo, Krzysztof Tomasiewicz

**Affiliations:** 1 Department of Medical Microbiology, Second Faculty of Medicine, Charles University and Motol University Hospital, 150 06 Prague, Czech Republic; pavel.drevinek@lfmotol.cuni.cz; 2 Czech Society for Medical Microbiology, Czech Medical Association of J.E. Purkyne, 142 20 Prague, Czech Republic; 3 Department of Infectious Diseases and Hepatology, Medical University of Bialystok, 15-540 Białystok, Poland; robert.flisiak1@gmail.com; 4 Polish Association of Epidemiologists and Infectiologists, 15-540 Białystok, Poland; krzysztof.tomasiewicz@umlub.pl; 5 Department of Preclinical Disciplines, Faculty of Medicine, Titu Maiorescu University, 040441 Bucharest, Romania; roxanamarianemes@gmail.com; 6 Romanian Society of Pneumonology, 050159 Bucharest, Romania; 7 Policy Wisdom LLC., Quebradillas 00678-2705, Puerto Rico; 8 Department and Clinic of Infectious Diseases and Hepatology SPSK-1, Medical University of Lublin, 20-081 Lublin, Poland

**Keywords:** acute respiratory infections, COVID-19, influenza, point-of-care testing, rapid testing, diagnostic testing, differential diagnosis, Europe, health policy

## Abstract

This review aims to explore the role of professional diagnostic rapid testing of acute respiratory infections (ARIs), especially COVID-19 and influenza, ensuring proper disease management and treatment in Europe, and particularly in Czech Republic, Poland, and Romania. The paper was constructed based on a review of scientific evidence and national and international policies and recommendations, as well as a process of validation by four experts. The development of new testing technologies, treatment options, and increased awareness of the negative multidimensional impact of ARI profiles transformed differential diagnosis into a tangible and desirable reality. This review covers the following topics: (1) the multidimensional impact of ARIs, (2) ARI rapid diagnostic testing platforms and their value, (3) the policy landscape, (4) challenges and barriers to implementation, and (5) a set of recommendations illustrating a path forward. The findings indicate that rapid diagnostic testing, including at the point of care (POC), can have a positive impact on case management, antimicrobial and antibiotic stewardship, epidemiological surveillance, and decision making. Integrating this strategy will require the commitment of governments and the international and academic communities, especially as we identified room for improvement in the access and expansion of POC rapid testing in the focus countries and the inclusion of rapid testing in relevant policies.

## 1. Scope and Methodology

This review aims to discuss and position the role and value of professional diagnostic rapid testing of acute respiratory infections (ARIs), particularly for coronavirus disease 2019 (COVID-19) and influenza, as a tool to ensure proper disease management and treatment in Europe. To achieve this, the document provides an overview of (1) the multidimensional impact of COVID-19 and influenza, (2) rapid testing and its value for differential diagnosis, (3) the policies and current recommendations on the management and diagnosis of COVID-19 and influenza, (4) the challenges and barriers to implementing professional rapid testing in a COVID-19 post-pandemic scenario, (5) and a set of recommendations illustrating a path forward to resolve, manage, or reduce the identified challenges and barriers. The recommendations build on the overviewed evidence and seek to support a broad range of stakeholders and decision makers as they navigate the transition to the endemic management of COVID-19, including governments, the academic community, health providers, and international organizations.

The methodology used to develop this document includes a review of the literature and scientific evidence, policies, and a process of validation and feedback with a group of four experts in relevant fields (microbiology, epidemiology, public health, pharmacology, and infectiology). Global, European, and country-level evidence from three focus countries—Czech Republic, Poland, and Romania—was collated and analyzed between 13 March and 7 July 2023. Peer-reviewed papers and official governmental and international organizations’ sources were prioritized, capturing the following dimensions:

- The multidimensional impact of ARIs (particularly COVID-19 and influenza) in Europe and the focus countries.
- Scientific perspectives and positions on the role and value of testing and differential diagnosis for the management of ARIs.
- National and international policy frameworks and recommendations on the management of ARIs (especially COVID-19 and influenza), including testing.

An overview of the main topics included (by dimension) and relevant sources can be found in Table 1.

The information was synthesized in a working document that was discussed, reviewed, and validated by experts during an online panel session held on 31 July 2023 and rounds of offline review. All participating experts approved the final document.

## 2. Background and Introduction

A novel coronavirus, later identified as the severe acute respiratory syndrome coronavirus 2 (SARS-CoV-2), was identified in China on 31 December 2019 [103]. The first case of COVID-19, the disease caused by this new virus, was identified in Europe in January 2020 [104] and between February and March in our focus countries (the Czech Republic, Romania, and Poland) [105,106,107]. As COVID-19 spread around the globe, on 11 March 2020, the World Health Organization (WHO) characterized COVID-19 as a global pandemic [103]. In July and October 2020, the European Medicines Agency (EMA) and the U.S. Food and Drug Administration (FDA) approved remdesivir, the first COVID-19 antiviral treatment [108,109]. With great expectations to put a halt to the pandemic, on 27 December 2020, vaccination began in Romania [110], the Czech Republic [111], and Poland [112]. The end of the emergency phase in the European Union (EU) was cleared by the European Commission in April 2022 [113], and by May 2023, the WHO finally declared an end to COVID-19 as a public health emergency [114].

As of 13 December 2023, 772,386,069 confirmed cases of COVID-19 had been reported along with 6,987,222 deaths, of which 277,379,680 cases and 2,257,825 deaths were recorded in the WHO European Region [8]. While Europe reports the highest number of cumulative cases in the world, the region with the highest number of total deaths is the Americas. Regarding our focus countries, the Czech Republic records the highest number of cases and deaths per million inhabitants [1,2], a figure that sits above the EU. And while Poland and Romania report a lower number of cases per million inhabitants than the one recorded for the region, they too have a higher mortality than the EU [1,2]. Similarly, regarding vaccination, the focus countries show a lower vaccination rate than the EU, both in terms of completion of the initial protocol and boosters (Table 2) [3,4].

The first human infection by the new strain of H1N1 virus was reported in the United States on 15 April 2009, and within the same month, the first case in Europe was identified [115,116]. By 11 June of the same year, the WHO characterized the outbreak as a pandemic, with efforts to develop a vaccine beginning soon after [114,115,116]. And by September, the FDA and EMA announced the approval of the first vaccine [116,117,118]. With immunization being one of the main strategies to contain the pandemic and prevent future outbreaks, between December 2009 and February 2010, a recommendation on annual vaccination (especially for high-risk groups) was made by the EU, European Centre for Disease Prevention and Control (ECDC), and Centers for Disease Control and Prevention (CDC) [115,116,119]. The WHO declared the end of the pandemic on 10 July 2010 [115,116]. The post-pandemic management of influenza is characterized by the introduction of policy frameworks as part of regular health practices and the prevention of future influenza pandemics. These frameworks or policies include the WHO Pandemic Influenza Preparedness Framework (introduced in 2011) [80], the introduction of influenza surveillance in Europe coordinated by the World Health Organization Regional Office for Europe (WHO/Europe) and ECDC (since 2014) [120], and the WHO Global Influenza Strategy 2019–2030 [78]. Since 2010, the WHO has published, twice a year, recommendations on vaccines, composed according to the prevalent circulating variants [121].

The WHO estimates that seasonal influenza results in 290,000 to 650,000 deaths each year globally [12], of which up to 72,000 might occur in the WHO European Region [13]. Regarding the focus countries, Romania and Poland have reported a mortality rate below the European Region average during the last ten years. In contrast, the Czech Republic has consistently reported a higher mortality rate than Poland, Romania, and the WHO European Region [14]. During the COVID-19 pandemic, and most notably at the onset, the reported burden of respiratory viruses, such as influenza and respiratory syncytial virus (RSV), declined globally [122,123]. According to the ECDC, influenza transmission in the EU/European Economic Area (EEA) countries was reportedly low between 2020 and 2021 as COVID-19 pandemic contention measures were implemented, and the following seasons (2021–2022 and 2022–2023) remained below pre-pandemic records [124]. The observed decline in activity of respiratory viruses (other than SARS-CoV-2) was undeniably multifactorial. On one hand, the observed reduction can be attributed to the implementation of pandemic control measures, including social distancing, restrictions on people’s mobility, and changes in testing priorities and surveillance efforts [123,125,126,127,128,129]. On the other hand, the observed reduction might also be a consequence of incorporating SARS-CoV-2 into the ecological niche of viruses, causing a displacement of other ARI viruses [130,131].

COVID-19 and influenza have also considerably impacted social and economic dimensions. During the pandemic phase of both diseases, dysfunctions in the healthcare sector led to excess mortality and worse health. These dysfunctions encompassed both hospitals—due to an overflow of emergency medical services, high intake of admissions, and staff shortages—and ongoing programs and services, such as screenings, immunization, and health services [11,18,19,23,24]. Dysfunctions in the healthcare sector observed during the influenza pandemic replicate yearly during influenza seasons [19,20]. Furthermore, evidence on mortality rates from the COVID-19 and influenza pandemics indicates a disproportional impact on socioeconomically disadvantaged groups [132,133,134]. These inequalities can be understood as a syndemic, arising from endemic inequalities in the social determinants of health [134].

In Europe, the COVID-19 pandemic has led to an increase in unemployment (recording a drop by 2% year over year in the EU) [11], economic contraction and limited progress on social indicators [9,11], and a drop in the exporting and importing of good and services [10]. While all EU industries were heavily affected by the pandemic, the extent to which they were affected, and thus the impact across countries, varied substantially [10]. During the influenza pandemic, an increased number of days called-in sick and loss of productivity was recorded, which continues to be one of the main socioeconomic concerns [18,19,20]. In Europe, influenza is responsible for approximately 10% of sickness-related absences from work [21]. The length of workplace absence in European countries is higher than in the United States, causing a substantial loss of productivity [22]. In the EU, costs of seasonal influenza are estimated to be EUR 6 to 14 billion annually [22].

It is also important to consider the implications of long COVID and other COVID-19 sequelae. According to the WHO, 36 million people across the European Region may have developed long COVID over the first three years of the pandemic [5]. Long COVID is a multidimensional disability affecting physical, mental, and cognitive health [6,7]. There is a need for a system to report long-COVID symptoms and multidisciplinary patient-centric research to increase understanding of the true burden and impact of the condition [7]. Long-flu or influenza sequelae also exist but are less frequent than long COVID [15]. Long-flu symptoms present similarly to long COVID, including anxiety, fatigue, pain, and breathing problems, among others [15,16,17]. In fact, the unveiling of long COVID has come alongside an increased understanding of long-term sequelae of other ARIs. For example, sequelae associated with RSV have also been documented in the literature [135,136].

Undeniably, many valuable lessons can be learned from the COVID-19 pandemic. It showed the importance of health and comprehensive health policies for sustainable development [23,24,25,26]. Moving forward, the world needs to work toward universal health coverage, strengthening health systems grounded in human rights, gender equality, and financial sustainability [25,27,28]. Governments need to build a new global pandemic agreement which recognizes global cooperation and coordination as an essential pillar and update their national pandemic preparedness plans to incorporate the use of non-pharmaceutical interventions [25,26]. And at the global level, surveillance and regulation should address domestic and wild animal trade and dangerous practices as a way of effectively preventing new pandemics [25,26,29]. An integrated approach for the prevention and preparedness of respiratory-virus-based pandemics and epidemics should be developed [28].

## 3. Overview of Rapid Testing Options

There are currently four primary types of tests available for ARIs: molecular tests, antigen tests, antibody tests, and viral culture tests. Test options also diverge based on whether they measure or detect single or multiple pathogens, with the latter known as multiplex tests. As we transition into the COVID-19 post-pandemic management of ARIs, testing will continue to have a central role. Moving forward, decision-makers will have to carefully consider the purpose of testing (whether it is for diagnostics, surveillance, or epidemiological) and resources to define which test to use, balancing test tradeoffs regarding accuracy, accessibility, affordability, broadness, and turnaround time [35]. In this section, we will first provide a brief overview of test options for COVID-19 and influenza to later reflect on their characteristics and eligibility for point-of-care (POC) diagnostic testing.

### 3.1. Types of Tests Available for Detecting ARIs

Using upper respiratory specimens, molecular tests can detect the genetic material (nucleic acids) of the virus. For COVID-19, the reverse transcription polymerase chain reaction (RT-qPCR) is the current reference diagnostic method [30]. While RT-qPCR is highly sensitive and specific tests, result turnaround times are approximately 24 h in conditions of limited logistic and laboratory capacity [30]. This is attributed to the conditions required for the storage of samples, specimens’ transportation, and need for laboratory facilities and trained professional to process samples [137,138]. However, there are also some rapid molecular options available that might improve their use in decentralized settings. In the case of influenza, rapid molecular tests can be applied by medical staff—not requiring the intervention of a laboratory technician—and yield results in 15 to 30 min [31,32,33,34]. For COVID-19, rapid options such as the loop-mediated isothermal amplification (LAMP) and the nicking enzyme-assisted reaction (NEAR) represent valuable rapid alternatives to the RT-qPCR. The LAMP and the NEAR (which we will discuss in more detail later) are two nucleic acid amplification techniques that have gained recent momentum as they might bring molecular testing closer to health practitioners and POC [42,43,44].

Antigen tests are used to diagnose acute infection by detecting viral proteins using upper respiratory specimens. For COVID-19, antigen tests are available for professional use and self-testing, providing results within 15 to 30 min [30]. Influenza antigen tests can be divided into two main groups: rapid antigen influenza diagnostic tests (RIDTs) and immunofluorescence antigen detection assays. Some RIDTs are approved for outpatient settings, while others require a moderately complex clinical laboratory. RIDTs have a clinically relevant turnaround time (10 to 15 min) and can differentiate between influenza types (A and B), but confirmation by molecular assays of negative results is recommended [32,33,34]. The immunofluorescence antigen detection test delivers results in approximately two to four hours and can also distinguish between influenza A and B [32,139].

Antibody tests detect antibodies generated against the virus from prior infection or vaccination. Since antibodies are detectable only a few weeks after acute infection, these tests are not recommended for diagnosis and clinical management, and instead are recommended for research or surveillance purposes [32,34,140]. Antibody tests are not able to differentiate antibodies for influenza A and influenza B in a single specimen, which limits its clinical value [32,139]. This is also the case with viral culture tests. Available only for influenza and other non-COVID-19 ARIs, viral culture tests have a lengthy turnaround time, with shell vial tissue culture taking one to three days and traditional tissue-cell viral culture three to ten days. Viral culture methods, however, allow for extensive antigenic and genetic characterization of influenza viruses, playing an essential role in the surveillance and characterization of new seasonal strains [32,141].

### 3.2. Overview of Rapid Test Options for POC Diagnosis of ARIs

POC rapid testing involves performing a test outside of laboratory conditions and closer to the patient’s location, seeking to improve access to the diagnosis of acute infections and, consequently, adequate disease management [142,143]. Thus, POC testing can be implemented in various decentralized settings, including at a primary healthcare facility, physician offices, urgent care facilities, pharmacies, school health clinics, long-term care facilities, nursing homes, and temporary locations, among others [144]. POC testing can be performed by healthcare practitioners, the individual being tested, or a family member or caregiver [144]. The use of POC testing can help (1) reduce barriers to care by enhancing decentralized, rapid, sensitive, and low-cost diagnosis, (2) improve timely disease identification and management (including access to targeted treatment and patients’ capacity to modify their behaviors), and (3) in parallel (while it is not the primary reason for POC use) contribute to surveillance and decision-making purposes by collecting real-world data [41,142,145,146]. The implementation of POC testing might be enabled by the presence of clear national policies, guidelines, and implementation plans; adequate funding; flexibility in the implementation model to meet different jurisdictional and health service needs; capacity building and training for healthcare workers; and robust quality control of tests, among others [65].

POC testing can be used to detect current infection by SARS-CoV-2, influenza, and other ARIs, such as RSV. Table 3 presents a summary of the tests available for POC, including a comparative analysis of their trade-offs in each case.

Antigen tests, available for professional use and self-testing, are a good alternative for POC and hospital settings, given their quick turnaround time; facility to implement, transport, process, and interpret; and low cost [31,35,36,41]. While RT-qPCR is the most common type of amplification technique used to diagnose COVID-19, its use for POC is limited due to its laboratory requirements. An effective molecular alternative for POC are isothermal amplification techniques [147], such as the LAMP and NEAR. These technologies are a potential game changer that can improve result turnaround times, ease the use of molecular diagnostic tools for COVID-19, and decrease the cost of testing (when compared to PCR-based options) [148,149,150]. Unlike RT-qPCR, isothermal amplification techniques require little to no training and can be performed at community health centers and hospitals by frontline workers like nurses and medical doctors [49]. 

The reverse transcription LAMP (RT-LAMP) is a viable alternative to RT-qPCR for POC due to its specificity and sensitivity (nears that of RT-qPCR), cost-effectiveness, minimal instrumentation needed (simplicity), and clinically relevant turnaround time [43,44,45,46,47]. However, this technology, like any other, has its limitations, mainly due to its risk of false positives [43,44], due to unintended primer cross-reactivity [47] or the pH-based colorimetry used in LAMP [43]. A typical LAMP test requires six primers targeting eight regions of the target sequence, which has been found to lead to high-level unintended primer cross-reactivity in the past [47]. More recent studies comparing RT-LAMP and RT-qPCR with matched samples have found that RT-LAMP reliably detects the virus in samples that amplify by RT-qPCR at a quantification cycle (Cq) < 30, reaching similar or better sensitivity than RT-qPCR under such conditions [44,45]. However, evidence also indicated that RT-LAMP is unlikely to identify the virus at concentrations that result in a Cq of 38 by RT-qPCR, which is a common cutoff point for positivity [44]. This risk of false positive results has been attributed to using pH dyes with crude clinical samples in RT-LAMP [44]. An RT-LAMP positive reaction can be determined visually by colorimetric or turbidity changes [47]. The pH of human saliva can vary from 6.8 to 7.4 [151], variability that can lead to a premature color change [152,153]. Multiple promising efforts to reduce this risk have been recorded in the literature, whether using a custom saliva stabilization solution or an alternative extraction method (nucleic acid extraction) [152,154,155,156,157].

NEAR is a novel automated technique with a very promising value for POC [43,50]. NEAR can achieve a linear amplification of a DNA template using two enzymes (nicking endonuclease and DNA polymerase) [158,159]. Polymerase activity was noted for improved sensitivity and efficiency in the past [49]. NEAR tests for COVID-19 can take place inside the manufacturer’s instrument (a closed automated system) using a qualitative fluorescence readout technique, requiring only the instrument and a cartridge to be applied [43]. Techniques of this nature have a reaction time of approximately five minutes (providing a real-time readout) due to the small size of the amplicon compared to other molecular tests, reducing the results turnaround time significantly [43,52]. Furthermore, NEAR tests can be adapted to different temperatures using different primers, polymerases, and nicking enzymes [43]. Nonetheless, among the disadvantages is the risk of false negatives at higher Cq values (usually above 35), in part because of the dilution due to the use of viral transport media prior to amplification (which is possible but not the preferred method) [43,53,160,161].

Aside from rapid single antigen and molecular assays, POC testing can be supported using rapid multiplex tests [59,60]. Multiplex testing allows for the simultaneous on-site detection of different analytes using a single specimen, which could mean saving time and resources [58]. By reducing incorrect or incomplete diagnosis of infectious diseases that have shared symptoms and clinical features [162,163,164], multiplex testing might help critical patients access much-needed treatment on time [32,139]. There are two main types of rapid multiplex tests currently available for ARIs: molecular and antigen [57,61]. Rapid multiplex PCR tests can include various combinations such as influenza A, influenza B, and SARS-CoV-2; influenza A, influenza B, RSV, and SARS-CoV-2; and up to 20 of the most common respiratory viruses and bacteria causing upper respiratory illness [54,61]. Rapid multiplex antigen tests have a more restrictive virus combination (including SARS-CoV-2, influenza A, and influenza B) [99,122] but are more easily implemented at the POC, requiring minimal training [35,36,37].

## 4. The Role and Value of Rapid Testing for the Management and Treatment of ARIs

The use of rapid diagnostic tests can contribute to the management of ARIs by removing diagnostic uncertainty, which in turn has at least three benefits, improving (1) case management, (2) antimicrobial and antibiotic stewardship, and (3) epidemiological surveillance and decision making. In this section, we will discuss the role and value of rapid diagnostic testing on these three fronts.

### 4.1. The Role and Value of Rapid Diagnostic Testing of ARIs for Case Management

The differential diagnosis of patients presenting respiratory symptoms due to bacterial or viral infections (including COVID-19 and influenza, among others) can enhance the clinical management of cases, offering critical information to provide adequate and timely treatment and care [31], thereby reducing the burden on hospital systems [31,65]. Evidence demonstrates that differential diagnosis can contribute to improving antiviral prescribing practices, with a particular positive effect on the management of high-risk patients whose prognoses vary depending on the virus [62,63,65]. Currently, there are three main classes of treatments for COVID-19: (1) direct-acting antiviral therapies, (2) monoclonal antibodies (mAbs), and (3) host-directed therapies. While the first and second are alternatives that can be used to treat mild-to-moderate cases in patients at a high risk of developing severe disease, host-directed therapies are reserved for treating hospitalized patients that require respiratory support [165,166,167,168,169,170]. Host-directed therapies for COVID-19 aim to dampen the dysregulated inflammatory response to severe COVID-19 disease, a manifestation that occurs later during infection when virus replication is typically past its peak [165,166].

In contrast, COVID-19 antivirals are most effective when administered early during infection, before the virus has reached its replication peak, preventing progression to severe disease [165]. In fact, the efficacy of antivirals was found to increase even with small reductions in time to treatment initiation [64,171]. According to current FDA guidelines, there are several approved antivirals: Nirmatrelvir with Ritonavir (Paxlovid) [172], Remdesivir (Veklury) [109], and Molnupiravir (Lagevrio) [170], all of which should be administered as soon as possible and begin within five (for Paxlovid and Lagevrio) to seven (for Veklury) days from symptom onset [170]. Similarly, in the case of influenza, evidence suggests that antivirals currently recommended for high-risk groups are most effective when taken within 48 h of symptoms onset [173,174,175]. The effective use of antivirals requires the timely rapid diagnosis of respiratory infections of high-risk patients [31,64], with the potential to expand to the general population in the near future [64].

Antiviral therapies are currently only recommended for patients at known risk of severe COVID-19 [176]. Nonetheless, some argue that in the future, antivirals could be used at an even broader scale to reduce recovery time and the potential long-lasting effects of symptomatic patients, as well as the onward transmission of the virus [64]. Whether this scenario will ever materialize, and for which individuals, is unclear. Though, in such cases, the availability of rapid diagnostics will be essential to bring the possible clinical benefits from direct-acting antivirals to even more patients [64].

### 4.2. The Role and Value of Rapid Diagnostic Testing of ARIs for Antimicrobial and Antibiotic Stewardship

Antiviral resistance is a natural phenomenon often caused by viral evolution. Nonetheless, evidence indicates an increase in drug resistance due to drug-induced selective pressure [66,177,178], propped by inappropriate use and excessive prescription [67,179]. The same applies to the use of antibiotics and development of antimicrobial resistance [68,180]. In particular, antimicrobial resistance has been associated with incorrect diagnoses, the overuse of medication due to concerns of bacterial co-infections, suboptimal antibiotic dosage in true bacterial infections, and the disregard for the harmful effects of unnecessary antibiotic treatment [65,68,180,181]. In the absence of a conclusive diagnosis, physicians often opt to prescribe antibiotics as a preventive rather than treatment measure [179]. Timely access to differential diagnosis might help improve patient treatment stewardship and, in turn, help reduce the risks of antibiotic resistance at individual and population levels [31,66,67]. A study found that in positive influenza cases, rapid diagnostic testing reduced unnecessary antibiotic prescriptions, and in negative cases, prompted the adequate treatment of bacterial infections [31].

In fact, in July 2022, the European Commission and the EU member states identified antimicrobial resistance as one of the top three priority health threats [182]. Approximately 35,000 people die each year in the EU/EEA as a direct consequence of antimicrobial resistance [183], and the health impact (burden of infections due to bacteria with antimicrobial resistance) is comparable to that of influenza, tuberculosis, and HIV/AIDS combined [68]. The cost to EU/EEA healthcare systems is approximately EUR 1.1 billion annually [68]. According to the European Commission, reducing avoidable antibiotic use can help mitigate the risks of resistance [184]. In this context, the use of rapid diagnostic testing is one of the primary interventions recommended by the Organization for Economic Co-operation and Development (OECD) and ECDC for reducing antimicrobial resistance [68].

European countries’ efforts to tackle antimicrobial resistance are guided by the WHO Global Action Plan on Antimicrobial Resistance, launched in 2015 [185], and the European Strategic Action Plan on Antibiotic Resistance, launched in 2017 [186]. The EU’s key objectives center around boosting the development of evidence, coordination, surveillance, and control measures. While most European countries have a national plan to combat antimicrobial resistance, these frameworks vary greatly in content and detail between countries [184].

### 4.3. The Role and Value of Rapid Diagnostic Testing of ARIs for Epidemiological Surveillance and Decision Making

As countries transition from COVID-19 emergency response to living with the virus, the role of diagnostic testing is shifting from the identification of cases and monitoring of outbreaks to surveillance purposes [187]. Surveillance efforts in this context have shifted from mass testing to the testing of highly exposed and at-risk groups, according to WHO recommendations [91,188]. Studies conducted during the emergency phase of the COVID-19 pandemic in hospital settings revealed that systematic screening using RT-qPCR tests of patients with respiratory symptoms can be an effective but highly resource-intensive containment strategy, unlikely to be sustained in the long run [69]. Nevertheless, as new rapid molecular testing technologies have become available, studies from high-resource countries, such as Germany, have shown promising results on the cost-effectiveness of such methods in emergency rooms and hospital settings [70,71]. Similarly, studies in Germany and Italy have also found economic benefits when using rapid antigen tests [72,73].

Data generated through POC testing, if recorded and reported correctly, can support ARI surveillance efforts, which in turn brings value to policy decision making to mitigate viral replication and spread [187]. These efforts might benefit from targeting specific population groups, instead of mass testing, according to the capacity and resources available in each context. By improving access to testing and reducing results turnaround times [65,74,146], decentralized rapid testing can help optimize infection control practices [65,74], raising alarms about unusual disease patterns or outbreaks [187], or offering reassurance that an incidence of a perceived health condition is not on the rise [75].

Continued investment in improving surveillance systems and data connectivity will ensure that physicians and decision-makers have the information needed to adequately care for patients and investigate outbreaks [35]. Furthermore, surveillance information gathered through diagnostic testing can support the management and implementation of health policies and programs [75]. Surveillance information gathered through POC rapid testing can help build rapidly reactive health systems [65,74,188], able to quantify the disease burden and monitor trends over time using real-world data. This in turn would allow for continued evaluation of measures taken and the planning of programs, services, or policies according to population needs [75].

The WHO, WHO/Europe, ECDC, and the European Surveillance Networks for COVID-19 and influenza recognize that there is an urgent need to establish and maintain resilient, population-based, integrated surveillance systems for influenza, COVID-19, and potentially other respiratory virus infections [55,76]. These systems should integrate sentinel-generated data as well as data generated through other forms of surveillance (e.g., event-based surveillance, non-sentinel surveillance, and mortality surveillance) [189]. In this context, increasing diagnostic testing capacity, utilizing appropriate technologies at every level, is crucial [187]. European countries are encouraged to continue strengthening their capacities for genomic surveillance and real-time data collection [55,190].

## 5. The Current Landscape of Policies and Recommendations on the Management of ARIs and Use of Rapid Testing

Having explored the role and value of rapid diagnostic testing for the management of ARIs, in this section, we provide an overview of the current global, regional, and national policy landscape, identifying the current recommendations and policy frameworks available for the management of ARIs, paying particular attention to COVID-19 and influenza, as well as those relevant to POC professional rapid diagnostic testing.

### 5.1. Policies and Recommendations Regarding the Management of ARIs in a Post-Pandemic Scenario

In 2023, the WHO launched a strategy seeking to support countries as they transition to the long-term management of diseases (2023–2025). This strategy aims to (1) reduce and control the incidence of COVID-19 variants, especially in high-risk and vulnerable population groups, (2) prevent, diagnose, and treat COVID-19 to reduce mortality, morbidity, and long-term sequelae, and (3) support countries’ transition to sustainable, integrated, longer-term COVID-19 disease management [76]. Similarly, at the regional level, WHO/Europe launched a guiding document proposing a paradigm shift for pandemic preparedness and response. The document presents actions in the areas of collaborative surveillance, community protection, clinical care, countermeasures, and coordination [81]. In 2022, the WHO endorsed the Global COVID-19 Vaccination Strategy, setting up the target to reach 100% immunization of healthcare workers, older populations (60+), and priority risk groups with primary series and booster doses, and 70% immunization of the total general population [77].

Regarding influenza, the WHO Global Influenza Strategy (2019–2030) seeks to increase vaccination coverage, reduce the seasonal influenza burden, minimize zoonotic influenza risks, and mitigate pandemic influenza’s impact [78]. With surveillance being an essential piece of the puzzle to achieve these goals, the WHO and WHO/Europe have introduced pandemic influenza preparedness frameworks, highlighting the importance of surveillance, health system preparedness, laboratory capacity, community engagement, and governance to adequately manage potential influenza pandemics [79,82]. And regarding immunization against influenza, the WHO, the ECDC, and the EU have all set a 75% immunization target for older age groups (75+) [78,119,191].

Regarding the policy landscape relevant to the management of ARIs in focus countries, we found that the Czech Republic, Poland, and Romania include respiratory illnesses in their current national health plans but do not specifically reference ARIs within these frameworks (Table 4) [83,85,87,192]. Instead, countries seem to manage ARIs only through independent policies. All three countries currently have a National Program for Influenza and a National Policy for Respiratory Illnesses (which have started to integrate COVID-19 among their efforts) [55,84,86,88]. However, in some cases, such as Romania, these policies are only focused on surveillance and fail to address the core aspects of disease management [88].

### 5.2. Current Recommendations for ARI Testing: COVID-19 and Influenza

Test development from the beginning of the COVID-19 pandemic was supported by international organizations which contributed to the distribution and validation of new technologies. The WHO was one of the first stakeholders to distribute COVID-19 test kits to countries and set up guidelines to develop COVID-19 tests globally [89]. Regardless of these efforts, in the European region, concerns arose regarding variations in the quality of tests according to different manufacturers [197]. To address this issue, the European Commission established a technical working group in charge of processing the application of new tests to be included in the EU common list [92,93]. With the end of the emergency phase, the technical working group was dissolved on 17 May 2023 [93]. Nonetheless, the European Commission will continue to monitor the development of new rapid molecular and antigen tests [94].

As of 1 August 2023, the WHO continues to recommend the use of both molecular and antigen tests for the diagnosis of COVID-19, prioritizing the use of antigen tests in settings where molecular testing capacity is limited [90]. Antigen tests are the current preferred diagnostic method for COVID-19 in the Czech Republic, Poland, and Romania [86,98,99,198]. Recommendations on the use of diagnostic tests in an endemic scenario have shifted to prioritized groups. According to the WHO, COVID-19 testing in this circumstance should transition to high-risk groups and individuals with moderate or severe symptoms [155]. The testing of asymptomatic individuals is no longer recommended unless for groups at risk who are highly exposed to infection [91]. For influenza, both at a global and regional level, diagnosis is presumed without laboratory testing when influenza is widespread in the community [34]. Applying laboratory diagnostic testing is required to differentiate influenza from other ARIs during periods of low influenza activity, outside of epidemic situations, and during syndemic periods (such as the tripledemic of 2022) [12]. Influenza testing is also recommended when it can help inform clinical management of cases, such as patients being admitted to the hospital [32].

At the regional level, the European Commission recommends prioritizing the use of rapid diagnostic methods, such as antigen tests, when there is a limited molecular testing capacity and where prolonged testing turnaround times result in no clinical value [94,95]. Since antigen test sensitivity is lower in settings or populations with low expected disease prevalence [199], the ECDC highlights the use of these tests in settings where SARS-CoV-2 prevalence is high [96,97]. Regarding the implementation of rapid diagnostic testing at the POC, both the WHO and ECDC consider molecular rapid tests a good alternative for community settings when performed by trained providers [90,96,97]. The ECDC recommends that sampling, testing, test analysis, and reporting be implemented by a trained healthcare or laboratory provider or a trained operator [96,97]. In the Czech Republic, Poland, and Romania, current recommendations consider both professional and self-testing options [98,100,101,102,198]. The reporting of results is an important component to support surveillance activities. The European Commission and ECDC call for countries to report test results to public health authorities and register them in data collection and reporting systems [94,96,97], ideally at local, regional, national, and international levels [96,97].

Regarding multiplex assays, the WHO, WHO/Europe, ECDC, CDC, and the European Commission all recognize the value of integrating multiplex testing for the diagnosis of ARIs [55,57,92,200,201,202]. According to WHO/Europe and ECDC, multiplex assays are the preferred diagnosis tool as they can reduce the use of reagents, needed consumables, and turnaround time [55]. Thus, where possible, specimens taken at primary and secondary care sentinel surveillance sites should be tested using multiplex PCR assays to simultaneously detect influenza, SARS-CoV-2, and other relevant respiratory viruses [55]. Antigen multiplex assays have been included in the EU common list of COVID-19 antigen tests, updated July 2023 [92]. And in the FDA’s list, only 1 of the 11 authorized multiplex assays for the simultaneous detection of influenza and SARS-CoV-2 is an antigen assay [57]. Although multiplex assays have great potential value, national guidelines in focus countries do not yet consider these technologies. Nonetheless, in certain cases, such as in Poland, combination antigen testing kits for influenza, RSV, and SARS-CoV-2 are being provided free of charge by family doctors [101]. And in the Czech Republic, the Society for Medical Microbiology has recommended the simultaneous molecular detection of influenza, RSV, and SARS-CoV-2 in patients with severe respiratory symptoms who require hospitalization, or in patients with risk factors [203].

## 6. Challenges and Barriers to Professional Rapid Testing of ARIs

Evidence indicates that diagnostic rapid testing for COVID-19 and influenza can play a positive role in the adequate management of ARIs in a post-COVID-19 pandemic scenario in Europe and our focus countries. However, the implementation of a successful policy or strategy for diagnostic rapid testing must consider and proactively include measures to reduce or mitigate potential challenges and barriers. Thus, in this section, we discuss the barriers and challenges to diagnostic rapid testing for COVID-19 and influenza. Challenges were identified based on the overviewed evidence, both from a policy and scientific perspective, and can be organized into three main groups: (1) challenges associated with intrinsic test limitations and characteristics, (2) challenges associated with the capacity to implement professional rapid testing strategies at the POC, and (3) challenges associated with the current policy frameworks and funding.

### 6.1. Challenges and Barriers Related to Intrinsic Test Limitations and Characteristics

Each type of diagnostic test has advantages and disadvantages. Antigen tests are considered the easier type of test to implement and use (as they are portable, less costly, require minimal training, and have a clinically relevant result turnaround time) [31,35,36,37,41], representing the preferred option for outpatient or emergency room settings [204]. However, antigen test sensitivity varies significantly between the types of assays, types of clinical specimens tested, the time of sampling after exposure, and the expected viral prevalence in the community [38,39,41]. And when compared to molecular tests, antigen test sensitivity is overall lower [37,40]. Less sensitivity means a higher risk of false negative results in people with low viral loads [38].

On the other hand, molecular tests are considered the gold standard for diagnosis due to their high sensitivity. However, they might be difficult to implement in decentralized healthcare settings (e.g., POC) and emergency rooms due to their general higher cost, longer turnaround time, and the conditions required for their application and processing (the type of equipment needed and sample storage requirements) [204]. While there are some rapid options available, such as NEAR and LAMP, which can detect infection in a reasonable timeframe (5 to 30 min) and can easily be implemented at POC [43,44,45,46,47,49,50,52], they also carry some limitations associated with intrinsic test characteristics (described earlier). This can lead to a risk of false positives (in the case of LAMP) [43,44] and false negatives (in the case of NEAR) [43,53]. While multiple promising efforts to reduce these risks have been made [152,154,155,156,157], more studies are urgently needed in order to validate these technologies for COVID-19 diagnosis [157]. Furthermore, for rapid molecular tests to be considered an acceptable option for diagnostic purposes at the POC and hospitals, research must demonstrate not only their sensitivity and specificity, but also how they perform against other factors that make up an ideal diagnostic test in such a setting. These factors include a rapid clinical decision, test safety and usage by non-laboratory staff, result turnaround times, and cost-effectiveness [49]. LAMP and NEAR have the potential to fulfil these criteria, but more studies are needed [49].

Rapid antibody tests have also been cited as an option for decentralized settings [144]; however, these tests are currently not considered a valid tool for diagnostics, and thus have limited value for POC. Serological tests have not been evaluated to assess protection levels [146]. The presence of antibodies captured by antibody tests cannot be equated to individuals’ immunity or active infection, which ultimately limits their clinical value [146].

While multiplex tests have been regarded as having a high clinical value, not all options available are eligible for POC settings [59,60]. Within eligible options, there are also some important tradeoffs to consider. Rapid multiplex PCR tests can detect a broader range of analyte combinates than rapid multiplex antigen tests but are generally more expensive [61]. On the other hand, rapid multiplex antigen tests can be more easily implemented at the POC with minimal training, but positive results require clinical correlation with patient history [35,36,37,54,55,57]. Furthermore, new multiplex test performance must be assessed across all known variants at the time of validation, which can be problematic as new COVID-19 mutations will likely continue to emerge [146].

### 6.2. Challenges Associated with the Capacity to Implement Professional Rapid Testing at the POC

One of the main challenges associated with the capacity to implement professional rapid testing in POC settings is the limited device availability and conditions to collect and process samples. According to reports from the European Commission on the state of health in Poland, Romania, and the Czech Republic, those countries record a lower testing capacity than the EU average due to a bottleneck in testing collection, sample processing, and device availability, which contribute to making testing temporarily inaccessible [198,205,206,207].

Health systems might also struggle with a limited availability of qualified personnel trained in various testing methods, competent to apply and interpret results [96,198]. Since the accuracy of rapid diagnostic tests largely depends on the conditions under which they are used, European countries need to guarantee the availability of training opportunities for healthcare providers to ensure adequate sampling, testing, interpreting, and reporting of results [96]. Besides basic notions on the interpretation of positive and negative rapid test results, the CDC identifies at least three considerations that can minimize being misled by false positive or false negative results: (1) collect specimens as early in the illness as possible, (2) follow manufacturers’ instructions, including acceptable specimens and handling, and (3) follow-up negative results with confirmatory tests as needed [41].

Challenges associated with implementing rapid diagnostic testing in non-hospital settings have also been identified. Molecular and antigen devices that have been authorized for the POC or at home require adequate sample collection to ensure the quality of results and avoid patient harm [65,96]. As mentioned earlier, factors such as proper storage, adequate sample collection, and accurate interpretation of the results are essential to obtain reliable results [41,65,96].

Furthermore, another barrier is the capacity to register test results and the availability and connectivity of information systems. The availability and connectivity of reported systems in the focus countries have been found to be insufficient in remote areas [65,198,205,206]. Underfunding and understaffing of the registry systems may also lead to poor-quality reports as communication links in the systems constrain pooling and consistency of data [205,208]. The execution of self-testing may increase the underreporting of cases (as it often results in poor-quality specimens), reduce the possibility of confirming results, and compromise the availability of valuable information to take action [38,96].

Finally, health-seeking behavior has also been identified as a challenge. People’s hesitancy to get tested has been reported in the focus countries [198,205,206]. Hesitancy was found to associate with factors such as individuals intending to avoid contention methods (e.g., mandatory quarantine), which may represent a potential loss of income.

### 6.3. Challenges Associated with Current Policy Frameworks and Funding

Currently, there is an absence of a clear and comprehensive policy that can introduce standards for POC professional rapid testing of ARIs [190]. Such a policy should ideally cover aspects beyond the implementation of the testing and encompass registry conditions, surveillance capacity, quality requirements, and reimbursement policies [190,209]. Reimbursement policies are necessary to define the conditions and requirements for the reimbursement of a test. This is, for example, the case in Romania, where free testing is possible with a physician’s referral, while voluntary testing must be paid for out of pocket [198].

As countries transition to the endemic management of COVID-19, a de-prioritization of testing, alongside other measures, is expected. It is uncertain that diagnosis will continue to be a public health priority in this new scenario. Thus, there is a need to demonstrate to decision-makers the value of differential diagnosis and the use of rapid testing and POC testing as a tool for the ongoing management of ARIs [146]. Changes in public health priorities, and therefore resources allocated to testing, will likely mean a shift in the accessibility of these tools. Testing needs to be strategically deployed to prevent access barriers to those who need them most [209].

Lastly, challenges regarding surveillance systems have also been identified. The existing influenza surveillance systems are not sufficiently sensitive and representative to enable joint COVID-19 surveillance in the focus countries. An effective surveillance system needs to be capable of associating variants with severity, comorbidities, age, and other risk factors [146,189,198,205,206]. Furthermore, integrating COVID-19 surveillance into influenza and other ARI surveillance systems will require improvements in data sharing and integrations between different surveillance platforms, as both sentinel and non-sentinel systems will be needed [189]. Challenges regarding the underfunding of registry systems will need to be addressed urgently to guarantee consistency in the data reported [205].

## 7. Recommendations to Advance Professional Rapid Testing of ARIs in Europe

Based on the evidence and the challenges and barriers identified, a set of 19 recommendations is provided to support the implementation of diagnostic rapid testing for COVID-19 and influenza in POC settings. These recommendations include actions that require immediate attention, as well as actions designed for the medium and longer term. The recommendations are grouped into four categories, of which the first three address the three groups of challenges and barriers discussed in the previous section.

- The first group includes recommendations to address knowledge gaps and develop evidence to reduce intrinsic test limitations (including the validation of new testing technologies for the POC).
- The second group consists of recommendations focusing on actions required to strengthen the capacity and ensure adequate implementation of POC testing strategies.
- The third group addresses the policy gaps for the inclusion of professional rapid testing in relevant policies.
- The fourth group includes recommendations to strengthen cooperation and coordination among different stakeholders.

### 7.1. Actions to Address Knowledge Gaps and Develop Evidence to Reduce Intrinsic Test Limitations

- There is a need to demonstrate the cost-effectiveness of POC rapid testing, whether it takes place at primary healthcare or in hospital settings. Research institutes and the academic community should endeavor to provide insights on the value of differential diagnostics of respiratory viral infections (especially COVID-19 and influenza) through professional rapid testing, considering the multidimensional socioeconomic impact. Testing platforms (including rapid multiplex assays) should be considered based on their benefits and limitations.
- Recommendations should be made to enhance the adequate use of testing options according to the activities, including disease management, surveillance, and public health policy decision making.
- Research institutes and the academic community, with the support of governments and the private sector, should continue to undertake efforts to resolve knowledge gaps and validate the use of new testing technologies (including rapid molecular tests) for POC diagnosis.
- Funding for the research and development of new tests should be prioritized. Research and development strategies need to consider performance verification and validation against potential future variants and tests’ added values, including prognosis.

### 7.2. Actions to Strengthen Capacity and Ensure Adequate Means of Implementation

- The focus countries should ensure that testing capacity is sufficient when needed (according to disease prevalence), including sample collection, storage conditions, test supply, and capacity for processing samples. The testing capacity needs to be aligned with a strategy to guarantee high-quality tests are available.
- Governments should develop and implement a strategy to battle testing hesitancy and testing overuse. The strategy should be based on an understanding of the causes of such behaviors and tackling structural conditions if necessary.
- Governments should implement and promote a training opportunity for healthcare workers to guarantee their capacity to implement rapid testing options at different levels of care, but especially at the POC. Training opportunities should have a wide scope, from the test application to its interpretation and reporting.
- The focus countries need to implement strategies to increase the available workforce, considering this was one of the main issues during the pandemic.
- Governments should ensure the connectivity of reporting systems, so information collected through professional rapid testing is integrated with a broader health information platform, enhancing the opportunity to use this evidence for policy decision making, and continue to understand the risk factors and health impact of COVID-19 and influenza.

### 7.3. Actions for the Inclusion of Professional Rapid Testing in Relevant Policies

- Governments should include clear guidelines concerning professional rapid testing in relevant respiratory infection policies. Guidelines should specify which test to use, in what setting, and for what purposes. Particular attention should be paid to the use of professional rapid tests at the POC. The guidelines should also provide information for the reimbursement of the tests.
- Given the risks of long COVID and COVID-19 and influenza-related sequelae, and factoring in the benefits of diagnosis for the clinical management of high-risk patients, antimicrobial and antibiotic stewardship, and hospital case management, governments should prioritize the differential diagnosis of ARIs (including COVID-19 and influenza), considering the prevalence of infection by prioritizing the testing of high-circulating viruses.
- The use of antigen or molecular rapid tests at the POC should be considered according to health system capacity (including laboratory and technical capacity), availability, resources and costs, and test characteristics (accuracy, accessibility, affordability, and result turnaround time).
- Governments should consider using professional rapid testing to support the monitoring of infection and disease and utilize this evidence for policymaking purposes. Evidence collected through testing can help monitor the burden of disease over time, control transmission, and prevent future outbreaks.
- Governments should allocate dedicated resources to implement a professional rapid diagnostic testing strategy for ARIs (including COVID-19 and influenza). The ability to ensure adequate implementation should be considered, in particular, workforce capacity, the procurement of high-quality tests, accessibility, availability, and research and development.
- Governments should provide regulatory standards for professional rapid testing. Regulatory standards will contribute to guaranteeing test results’ accuracy, test quality, and proper implementation.

### 7.4. Actions to Strengthen Cooperation and Coordination among Different Stakeholders

- International organizations and professional societies should provide technical cooperation and support the development of national guidelines concerning the use of professional rapid tests across different settings and conditions. These guidelines should be based on international/regional standards.
- International organizations and professional societies should provide guidance and support to national decision-makers regarding the use of professional rapid tests in different settings, particularly at the POC.
- Policymakers, medical societies, the private sector, and academic communities should form a cross-functional partnership to collaborate on the ongoing expansion of knowledge and skills related to respiratory infection diagnostics. Emerging information should be shared freely and globally.
- Existing epidemic and pandemic response and preparedness plans (at national and international levels) should include multistakeholder-developed strategies to strengthen healthcare systems, improve market sustainability, and integrate differential diagnostics.

## 8. Conclusions

The development of new testing technologies and increased awareness of the negative impact respiratory viruses have on people’s lives, the economy, and health systems, prompted by the COVID-19 pandemic, renders decentralized differential diagnosis of ARIs a tangible and desirable reality. This study aimed to explore the role and value of this strategy as a tool to ensure proper disease management and treatment, considering existing evidence and the reality of European countries, particularly in the Czech Republic, Poland, and Romania. A scoping review published by the ECDC in 2022 regarding POC testing for the surveillance, prevention, and control of infectious diseases called for additional studies to better understand the types and characteristics of available POC tests, especially for COVID-19, recognizing the value of this strategy in Europe [210]. In this paper, we provided an overview of the test technologies currently available for COVID-19 and influenza, paying particular attention to rapid platforms, considering their advantages, intrinsic test limitations, and characteristics, and how they relate to their use at the POC.

Our analysis revealed that the Czech Republic, Poland, and Romania currently favor antigen tests as their preferred diagnostic method for COVID-19, considering both professional and self-testing options. This approach aligns with international organizations’ recommendations on the utilization of both molecular and antigen tests for diagnosing ARIs, considering countries’ testing capacity [35]. Our findings suggest the need to improve implementation and access to the different rapid testing options, a topic that has been the subject of discussion in previous studies [31,65,146]. Yet, rapid POC diagnostic tests are rarely used in most European countries [211]. Policies addressing ARIs can facilitate implementation, expansion, and improvements in POC rapid testing. Updating policy frameworks and guidelines might be considered in light of the evidence provided in this paper, according to national priorities and resources.

The implementation of molecular tests can be hampered by the need for the specific training of healthcare providers and adequate infrastructure [212,213], while new rapid options such as NEAR and LAMP (especially automated formats) might be viable alternatives [43,44,45,46,47,50]. Multiplex tests might provide a more accurate diagnosis using a single sample and subsequently save valuable time in accessing life-saving treatment in severe or high-risk cases [214,215,216], meaning these tests are noteworthy options for hospital settings [61]. However, our analysis revealed that multiplex testing is currently not considered in the national guidelines in focus countries. While guidelines and policies need to be updated considering all available options, today, rapid antigen and rapid molecular tests represent the best alternatives for the POC.

Decisions on the use of rapid molecular and rapid antigen options, or the combination of both, should consider cost-effectiveness, budget impact and feasibility, testing demand, capacity to integrate testing processes into the clinical workflow at different levels of care, treatment pathways, test accuracy, and testing turnaround times [217,218]. This is particularly important as new rapid molecular and multiplex technologies become available. In low-resource countries, rapid antigen tests have shown to be a cost-effective strategy which has increased timely access to diagnosis, regardless of epidemic phase [219,220,221]. On the other hand, rapid molecular and multiplex tests, which in general are more expensive than RT-qPCR, show promising cost-effectiveness in studies undertaken in emergency rooms and hospital settings in high-resource countries [70,71]. Whatever the option, accurate diagnosis remains critical as effective treatments for COVID-19 have the potential to offer value for money to healthcare systems, people, and society [222].

In this post-pandemic context, it is unclear if the diagnosis of ARIs will continue to be a public health priority, although robust testing has been recognized as an essential part of developing a more resilient healthcare system in Europe [223]. This is concerning, as this review did not find robust evidence of increased use of rapid testing for ARIs in the year following the COVID-19 pandemic. Emphasizing the value of differential diagnosis and leveraging rapid POC testing become crucial for effectively managing ARIs. Integrating a testing strategy for decentralized professional diagnosis encompassing POC rapid tests will require the commitment of governments and the international and academic communities to resolve, reduce, and mitigate persistent challenges and barriers.

The recommendations outlined in this review delineate some of the steps that key stakeholders can adopt to foster the adequate utilization and implementation of rapid professional diagnostic testing of ARIs, particularly in decentralized settings. Measures required by these steps require fostering research and knowledge advancement, facilitating evidence-based decision making, strengthening the capacity of health systems and healthcare providers, and deploying policy changes. Effective collaboration and coordination among diverse stakeholders, encompassing public and private sectors, the scientific and academic community, and governments, are indispensable to ensure the region and focus countries harness the fullest potential of available technological tools to optimize health outcomes for the general public and especially for critical population groups.

## Figures and Tables

**Table 1:** Dimensions and topics assessed in the literature and policies.

Dimension of Analysis	Topic	Key Sources
The multidimensional impact of ARIs	COVID-19	Health impact [1,2,3,4,5,6,7,8] Socioeconomic impact [9,10,11]
	Influenza	Health impact [12,13,14,15,16,17] Socioeconomic impact [18,19,20,21,22]
	Lessons learned from the COVID-19 pandemic	[23,24,25,26,27,28,29]
The role and value of testing and differential diagnosis for the management of ARIs	Testing platforms	COVID-19 [30] Influenza [31,32,33,34]
	The characteristics of rapid antigen tests	COVID-19 [35,36,37,38,39,40] Influenza [32,37,41]
	The characteristics of rapid molecular tests	LAMP [42,43,44,45,46,47,48,49] NEAR [43,49,50,51,52,53]
	The characteristics of multiplex tests	Antigen multiplex [35,36,37,54,55,56,57] Molecular multiplex [37,54,58,59,60,61]
	The value of differential diagnosis	Clinical management [31,62,63,64] Health systems [31,62,63,65] Drug resistance [31,66,67,68] Surveillance [69,70,71,72,73,74,75]
National and international policy frameworks on the management and testing of ARIs	Frameworks for ARI management	Global [76,77,78,79,80] Europe [79,81,82] Czech Republic [83,84] Poland [85,86] Romania [87,88]
	Frameworks for ARI testing	Global [34,56,69,89,90,91] Europe [34,55,92,93,94,95,96,97] Czech Republic [98,99] Poland [86,100,101] Romania [102]

LAMP: loop-mediated isothermal amplification; NEAR: nicking enzyme-assisted reaction. Source: elaborated by authors based on overviewed evidence.

**Table 2:** Impact of COVID-19 in the world, EU, and focus countries (Poland, Czech Republic, and Romania).

Country/ Region	Total Cases Reported per Million Inhabitants (as of 6 December 2023) [2]	Total Deaths Reported per Million Inhabitants (as of 6 December 2023) [1]	Share of People with a Complete Initial Vaccination Protocol Relative to Population (as of 14 September 2023) [3]	Vaccine Boosters Administered per 100 People (as of 14 September 2023) [4]
World	96,818.54	875.97	64.85%	35.06
EU	410,059.65	2774.03	72.86%	62.10
Czech Republic	446,372.92	4099.39	65.69%	49.25
Poland	164,420.14	3004.55	56.83%	38.61
Romania	178,093.74	3488.94	41.28% ^a^	-

^a^ evidence according to latest available data as of 11 June 2022. Source: elaborated by authors based on overviewed evidence.

**Table 3:** Types and characteristics of POC rapid tests for COVID-19 and influenza.

Types of POC Rapid Diagnostic Tests	Advantages	Disadvantages
Rapid antigen tests	Results within 15 to 20 min [31,35,36,41]. Portable and easy to perform [31,36,41]. Less costly than laboratory tests [35,36]. Implementation requires minimal training [31,35,36,41]. Cheaper and faster to manufacture than molecular tests [35].	Not as sensitive as molecular tests or viral culture [31,35,41]. Varying sensitivity. The sensitivity to detect influenza B is lower than for influenza A [41]. Narrow range of targets (some tests do not distinguish between influenza A or B or virus subtypes) [31,41]. Risk of false positive and false negative results when virus prevalence is low or high, respectively [41]. Positive results require confirmation in low-prevalence settings [35].
LAMP (rapid molecular tests)	Results within 30 min [43,45]. Specificity and sensitivity close to RT-qPCR [44,46]. Cost-effective [43,45]. Minimal instrumentation needed to perform test [43,45].	New assays are difficult to design [43,47]. Risk of false positives due to unintended primer cross-reactivity 90 or the pH-based colorimetry [43]. Might require refrigeration and more strict control measures than RT-qPCR [43,48].
NEAR (rapid molecular tests)	Results within 5 minutes for positive results and 15 min for negative results [43,50,51,52]. Easy to implement in decentralized settings as it uses closed systems [43,50,51]. Adapts better to different temperatures [43,50,51].	Risk of false negatives associated with the dilution due to the use of viral transport media [43,53].
Rapid multiplex PCR (molecular multiplex)	Stand-alone method for diagnosis, with great value for severely immunosuppressed patients [37,58]. Detects a broad range of combinations, including the most common respiratory viruses and bacteria causing upper respiratory illness [54,61]. Provides evidence on viral strains [37]. Can be used for burden of disease and virus surveillance [37].	Only a few can be used at the POC [58]. Requires certified laboratories to perform high-complexity tests [58].
Rapid multiplex antigen (antigen multiplex)	Easily implemented at the POC as it requires minimal training [35,36,55]. No laboratory requirements [35,36,55]. Detects SARS-CoV-2 and influenza A and B [54,56] Can be used for burden of disease surveillance [35,36,55].	Positive results need clinical correlation with patient history to determine infection status [35,36,55].

Source: elaborated by authors based on overviewed evidence.

**Table 4:** National policy frameworks for the management of respiratory illnesses and ARIs in focus countries: Czech Republic, Poland, and Romania.

Country/Region	National Health Plan Includes Respiratory Illnesses	National Health Plan Includes ARIs	National Policy Program for Influenza	National Policy for Respiratory Illnesses (NPRI)	Does the NPRI Integrate COVID-19 and ARIs?
Europe	Yes [192]	Yes [192]	Yes [193,194]	Yes [55]	Yes ^a^ [55]
Czech Republic	Yes [83]	No [83]	Yes [195]	Yes [84]	Yes [86]
Poland	Yes [85]	No	Yes [86]	Partial ^b^ [86]	Yes [86]
Romania	Yes [87]	No	Yes [196]	Yes [88]	Yes ^a^ [88]

^a^ specific for surveillance. ^b^ only for influenza and COVID-19. Source: elaborated by authors based on available data from official governmental sources (Ministry of Health, National Health Institutes, departments of surveillance, national regulatory agencies).

## Data Availability

Not applicable.
